# Hearables: feasibility of recording cardiac rhythms from head and in-ear locations

**DOI:** 10.1098/rsos.171214

**Published:** 2017-11-15

**Authors:** Wilhelm von Rosenberg, Theerasak Chanwimalueang, Valentin Goverdovsky, Nicholas S. Peters, Christos Papavassiliou, Danilo P. Mandic

**Affiliations:** 1 Department of Electrical and Electronic Engineering, Imperial College London, London, UK; 2 ElectroCardioMaths Programme, Myocardial Function Section, Imperial College and Imperial NHS Trust, London, UK

**Keywords:** wearable health, electrocardiogram, head-ECG

## Abstract

Mobile technologies for the recording of vital signs and neural signals are envisaged to underpin the operation of future health services. For practical purposes, unobtrusive devices are favoured, such as those embedded in a helmet or incorporated onto an earplug. However, these locations have so far been underexplored, as the comparably narrow neck impedes the propagation of vital signals from the torso to the head surface. To establish the principles behind electrocardiogram (ECG) recordings from head and ear locations, we first introduce a realistic three-dimensional biophysics model for the propagation of cardiac electric potentials to the head surface, which demonstrates the feasibility of head-ECG recordings. Next, the proposed biophysics propagation model is verified over comprehensive real-world experiments based on head- and in-ear-ECG measurements. It is shown both that the proposed model is an excellent match for the recordings, and that the quality of head- and ear-ECG is sufficient for a reliable identification of the timing and shape of the characteristic P-, Q-, R-, S- and T-waves within the cardiac cycle. This opens up a range of new possibilities in the identification and management of heart conditions, such as myocardial infarction and atrial fibrillation, based on 24/7 continuous in-ear measurements. The study therefore paves the way for the incorporation of the cardiac modality into future ‘hearables’, unobtrusive devices for health monitoring.

## 1. Introduction

We are witnessing a rapid development of wearable devices for the measurement of vital signs and neural signals, for both medical and recreational purposes. For user convenience, most suitable are inconspicuous and discreet devices, or those which make use of the appliances or clothing already worn by the user. It therefore comes as no surprise that state-of-the-art wearable devices for recording cardiac signals are mainly in the form of: (i) wrist bands, whereby the heart rate is typically recorded through the photoplethysmogram (PPG), or (ii) chest straps which record the standard electrocardiogram (ECG) [1]. However, PPG is suitable only for measuring the heart rate, and recording ECG from the wrists would require cables running between the arms, while chest straps can be obtrusive and stigmatizing, which makes these current solutions compromised in real-world applications.

To this end, we here consider the head as a location for placing wearable sensors, as the head is in a relatively stable position with respect to the vital signs in most daily activities, such as sitting, walking or sleeping. In our recent work, we have proposed a smart helmet with embedded sensors to record cardiac and neural signals in real-world scenarios from various locations on the head [2–4]. The feasibility of reliably extracting the timing of QRS-complexes, together with the detection of standard neural responses from the face-lead electroencephalogram (EEG), was demonstrated through extensive experimentation. However, despite success of this proof-of-concept, through experimentation only it is not possible to evaluate the sensitivity with respect to every factor that affects its quality. For example, for the head-ECG recordings, the signal quality in our experiments was strongly dependent on: (i) the positioning of the electrodes and (ii) the skin–electrode contact.

Furthermore, it is not uncommon to draw misleading conclusions from only empirical evidence of a phenomenon. This is particularly critical for weak data where the existing models are not applicable and the experimental evidence is only emerging, such as our considered head- and ear-ECG scenarios. Indeed, when it comes to head- and ear-ECG, both the biophysics model for the propagation of cardiac electric signals from the heart to the head, feasibility of medical quality ECG from head locations and a comprehensive experimental evidence have been missing, and are a subject of this study.

Early models for the simulation of the cardiac electric potentials on the body surface (body surface ECG) were based on idealized body geometries, such as a single homogeneous sphere [5], which unrealistically assumes constant dielectric parameters across the body. For enhanced accuracy, more advanced models typically consider a geometry consisting of two shells with realistic geometries, whereby one shell represents the heart muscle with the other shell representing the surrounding homogeneous torso [6,7]. However, to date there are no available biosignal propagation models which include both properties of the tissues along the propagation path of cardiac signals and realistic body geometries of the limbs, head or ear canals.

One aim of this work is therefore to provide a rigorous theoretical justification for the feasibility of a quality head-ECG. This is achieved through the introduction of a biophysics model of the propagation of cardiac potentials to the head surface, based on simulations of electric currents in the heart muscle and the examination of the resulting electric potentials on the body surface. The dielectric properties of the tissues along the propagation path, from the heart to a variety of head locations, are taken into account and include, among others, muscle tissue, lungs, bones, blood, skin and brain tissues. A good match between the simulations and measurements would mean that no artefacts or undesired signal sources are present.

The existing studies on recording ECG from head locations [2–4,8] focused solely on identifying the timings of R-waves in the signal. This limits their practical usefulness to only monitoring the heart rate, without the possibility of recording a full cardiac cycle which includes the timing and shape of the P-, Q-, S- and T-waves. To this end, to support our early experimental findings in [2–4], we here introduce a new comprehensive model for the propagation of cardiac electric potentials to the head surface (head-ECG), with the focus on identifying the extent to which the components of the ECG can be reliably recorded from several convenient locations on the head. This gives a theoretical background for both the feasibility of recordings from helmets (e.g. a smart helmet) and devices worn around the ear [8] or in both ears [9,10], so-called ‘hearables’ [11].

Given the stable location of the head and the ear canal relative to essential organs when sitting, walking or sleeping, head- and ear-worn devices are envisaged to play a major part in future wearable health.

Our findings demonstrate the ability of our head- and ear-based ECG set-ups to record cardiac cycles, the shapes of which are shown to be very similar to Lead I from the standard *limb leads*. In other words, the proposed head-ECG framework promises to enable the examination of heart conditions that are visible in multiple consecutive cardiac cycles in Lead I. The conditions include, among others, myocardial infarction (reflected in an elevated ST segment), first-degree atrioventricular block (the PR interval is longer than 200 ms), atrial fibrillation (the P-wave disappears, found in 2% to 3% of the population in Europe and the USA [12]), sinus tachycardia (elevated regular heart rate, P-wave can be close to the preceding T-wave) and atrial flutter (atria contract at up to 300 bpm, atrioventricular node contracts at 180 bpm, frequency of P-waves is much higher than the frequency of QRS-complexes) [13–15]. More generally, the proposed head-ECG framework paves the way for 24/7 continuous and unobtrusive cardiac monitoring and can alert the user when universal signatures of heart malfunction, such as a deep Q-wave or an inverted T-wave, are observed.

The results of this study therefore open up a new perspective for numerous existing applications in the community, such as an insight into the activity of the autonomic nervous system and its components, the parasympathetic nervous system and sympathetic nervous system [16,17], and an early-warning and tele-monitoring system for certain cardiovascular diseases.

## 2. Material, methods and set-up

### 2.1 Model geometry

**Figure 1.**
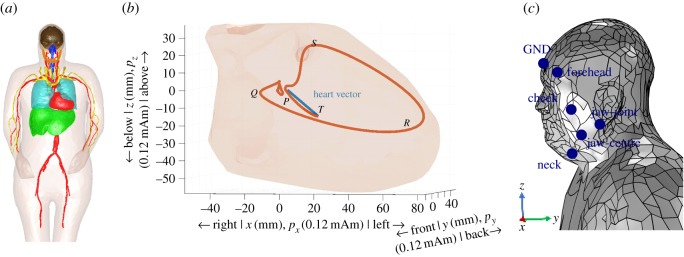
The computational biophysics model and experimental electrode positions. (*a*) *Body geometry*: Organs around the heart and in the head are inserted into a realistic full-body three-dimensional shell [18]. (*b*) *Heart vector*: The orientation and magnitude of the current dipole *p* in *p*_*x*_, *p*_*y*_, and *p*_*z*_; the heart vector at one point in time is shown in blue, and the trace of the tip of the heart vector from the start of the cycle until the current position (axes in 0.12 mAm) is shown in orange; the heart muscle is shown in pink in the background (axes in millimetres) (*c*) *Head-ECG set-up*: Symmetric set-up across the sagittal plane with electrodes on both sides of the head with the left side locations shown in blue, that is, the left neck, jaw-centre, jaw-joint, cheek and forehead locations.

Our feasibility analysis is based on a novel three-dimensional model, for which the body geometries are taken from the realistic VHP-Female Computational Phantom v. 2.1 and v. 2.2 [18]. The phantom was adjusted manually in instances when computational problems were experienced with the mesh of the model structures. The models of a complete body shell and the organs around the heart and inside the head were imported into the COMSOL Multiphysics^®^ software [19], and the upper part of the so-obtained torso and head model is shown in figure 1*a*). Finally, the whole body was surrounded by a sphere of radius 3.3 m filled with air, the complete mesh consisted of 560 630 domain elements and 72 286 boundary elements, and the average edge length was 6 mm.

### 2.2 Electric properties of the body and cardiac current sources

The values for the electrical conductivity and relative permittivity—a factor describing how the force between two static electrically charged particles is reduced when the vacuum between the charges is replaced by a given material—of the different body parts were taken from [20], for oscillations at a frequency of 15 Hz, and are presented in table 1, while the volume around the organs was filled with muscle tissue.

**Table 1.** Body tissues in the proposed realistic body model, and their dielectric properties at 15 Hz [20].

tissue	rel. permittivity	elec. conductivity (S m^−1^)
blood	5.26×10^3^	0.7
bone, cortical	3.59×10^4^	2.00×10^−2^
cerebrospinal fluid	109	2
grey matter	3.62×10^7^	3.50×10^−2^
heart	2.16×10^7^	5.76×10^−2^
liver	9.92×10^6^	3.08×10^−2^
lung, inflated	2.58×10^7^	4.60×10^−2^
muscle	2.51×10^7^	0.204
air	1	0

Previous research suggests that the simulation results for body surface ECG resulting from an isotropic model of the heart do not differ significantly from the results obtained based on an anisotropic structure of the heart model [21], and we therefore used an isotropic architecture. Also, owing to the complex morphology of the heart, the electric potentials on the torso surface are best modelled using multiple underlying dipole sources [22]. However, the head surface is relatively far away from the heart muscle (compared with the size of the heart) and thus admits the usage of a single dipole source, as adopted in this study. The orientation and magnitude of the current dipole, the heart vector, and its dynamics during the cardiac cycle are shown in figure 1*b* and further elaborated in [23]. The current dipole in this study was constructed by superimposing three orthogonal current dipoles—in the sagittal, coronal and transverse planes—with the amplitude variations in each dimension modelled based on real-world multi-lead ECG measurements on a human without known cardiac abnormalities, Subject 1 in this study. The time window in the analysis started 200 ms before and ended 400 ms after the maximum of the R-wave within the cardiac cycle.

### 2.3 Electrode arrangement

**Figure 2.**
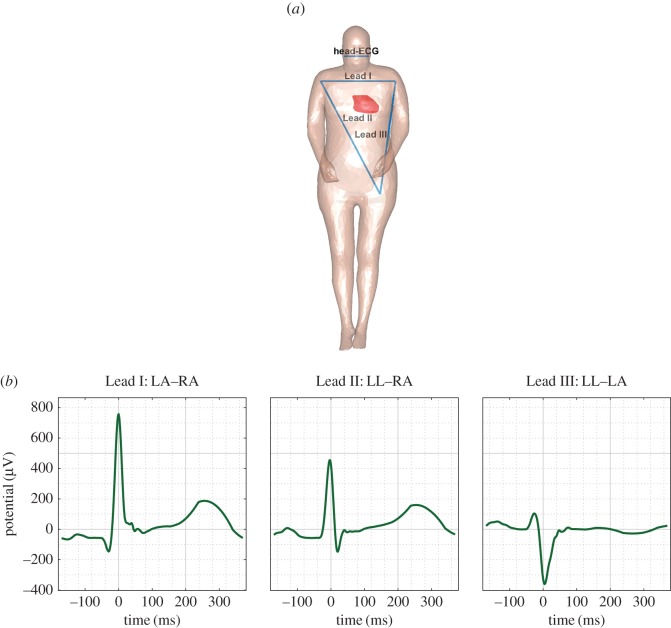
Simulated cardiac cycles for the *limb leads*. (*a*) The electrode positions for the three *limb leads* and head-ECG. (*b*) The simulated cardiac cycles for the well-known limb lead positions; these are a good match to the shape of the limb lead cycles reported in the literature [23].

The electrodes for the so-introduced *head leads* in the simulation and the experiments were attached symmetrically across the sagittal plane, and the locations included both sides of the neck (under the strap of a motorcycle helmet), the centre of the left and right parts of the lower jaw, the jaw-joints, the cheeks and both sides of the forehead (figure 1*c*). The ground electrode (GND) was placed in the middle of the forehead. For practical relevance, all head locations were chosen so that the electrodes could be attached to the lining of a motorcycle helmet at positions where the lining firmly touches the head [17]. As a reference, we used Lead I (figure 2*a*) from the standard *limb leads* ECG set-up from the arms. All head-ECG channels were recorded using standard gold cup electrodes (10 mm diameter) and conductive gel. The electrodes were connected to an Avatar biosignal recorder (manufactured by EGI) in a bipolar arrangement and sampled at a frequency of 500 Hz, where the reference electrode of each ECG channel was on the right half of the body. For validation purposes, additional virtual sensing positions were added to the simulation model, to model cardiac cycles from all three leads of the standard *limb leads* ECG, another important step in a comprehensive cross-validation between the proposed simulation model and real-world recordings.

### 2.4 Signal processing

The above-described set-up was tested over six subjects whose ECG were monitored for 180 s, so that the recorded time series contained between 167 and 245 heartbeats (excluding the very first and last cardiac cycle in order to consider full cycles only). The processing steps for the recorded ECG signals are explained in algorithm 1, and consisted of the following procedures. For the R-wave extraction, the procedure outlined in [24] was applied to the reference channel and the algorithm introduced in [4] was applied to each head channel separately. The latter algorithm uses a third-order bandpass filter with a lower cut-off frequency of $f_{\min}=9\;\mathrm{Hz}$ and an upper cut-off frequency of $f_{\max}=28\;\mathrm{Hz}$, applied to raw ECG before the R-waves were extracted. The timings of the identified R-waves in all channels and the original ECG traces were used as input to a new function, which bandpass-filtered all traces using a third-order bandpass filter with a lower cut-off frequency of $f_{\min}=3\;\mathrm{Hz}$ and an upper cut-off frequency of $f_{\max}=40\;\mathrm{Hz}$. Subsequently, the sampling points in time windows around the QRS-complexes, starting 250 ms before and ending 400 ms after the ‘peaks’ of the R-waves, were extracted in two different ways, using the R-wave timings as identified in: (i) the reference ECG from the arms (Lead I) and (ii) the actual head-ECG channel under consideration.

The cardiac cycle in each ECG channel was estimated by averaging the data segments across several consecutive time windows. This results in one average cycle per channel and the R-wave extraction method (i) using the R-wave timings from the reference channel and (ii) using the R-wave timings from the head-ECG channel itself. Owing to less evident QRS-complexes in the head channels, compared to standard electrode locations, there were some misidentified R-wave timings. Those misidentified R-wave timings resulted in differences in the averaged cardiac cycle depending on whether the R-waves (which were used as reference points during the averaging) were identified using the reference ECG from the arms (Lead I) or the ECG from the individual head channels.

For the head-ECG channels for which the averaged cardiac cycle corresponded to the expected cycle when the R-wave timings were detected using the reference ECG, this indicated that sufficient information about the cardiac cycle was present in the channel under consideration, i.e. the electrode locations are suitable to record the features of the cardiac cycle. When this was fulfilled for a head-ECG channel under consideration but not when the head-ECG channel itself was used to obtain the R-wave timings, a lack of good correspondence with the expected cardiac cycle indicated that the signal-to-noise ratio (SNR) in the given channel was too low for a reliable QRS-complex detection. However, an improvement of the QRS-complex detection algorithm or of the SNR would enhance the matching between the recorded and the expected cardiac cycle.

Parts of the here presented apparatus and methods have been filed to the UK Intellectual Property Office [25]. The scripts for the data analysis and the obtained data files have been deposited at Dryad: https://doi.org/10.5061/dryad.3n08c.


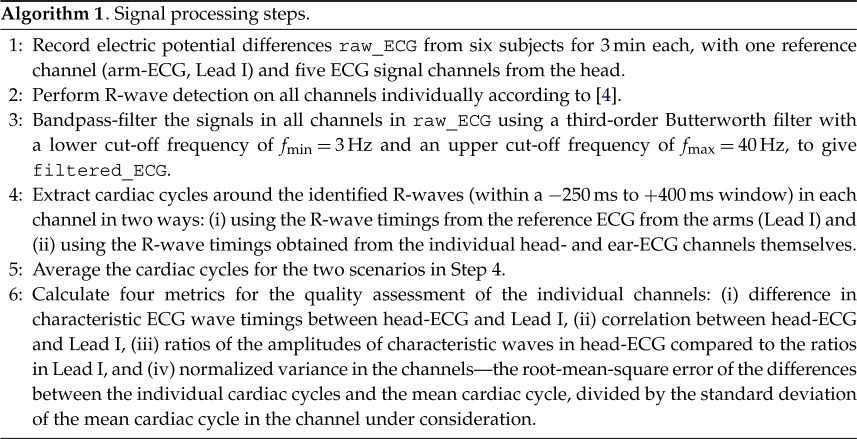


## 3. Results

**Figure 3.**
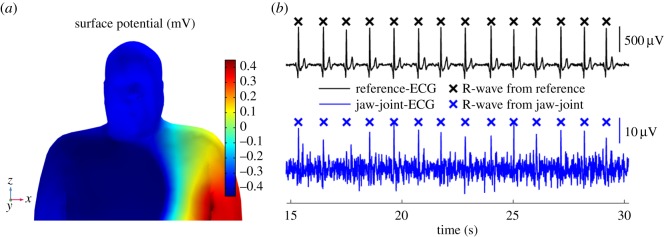
Simulated and recorded cardiac electric potentials on the upper torso and head surface. (*a*) Simulated electric potentials on the head surface and upper torso at the time of the R-wave peak (in millivolts). (*b*) Recorded ECG traces from head positions (ii, in blue) compared to standard Lead I from the arms (i, in black).

**Figure 4.**
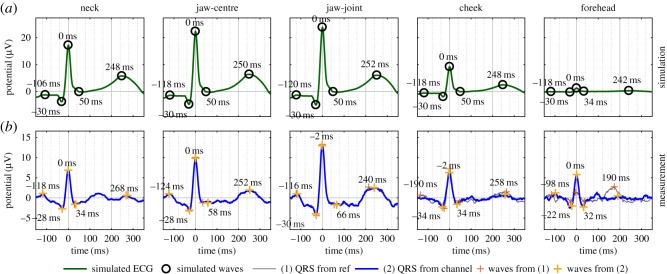
Simulated and measured cardiac cycles in five head-ECG channels for Subject 1. (*a*) Simulated cardiac cycle for the considered electrode positions. (*b*) Mean cardiac cycle for the corresponding electrode pairs of the recorded head-ECG. The cardiac cycles were identified based on (1) the timings of the R-waves in the arm ECG (grey lines with orange ‘+’) and (2) the timings of the R-waves detected in the individual head-ECG channels (blue lines with yellow ‘+’).

The forward model for the propagation of cardiac surface potentials was computed for a time window starting 200 ms before and ending 400 ms after the maximum of the R-wave in the input heart vector. Figure 3*a* shows a snapshot of the so-simulated cardiac potentials on the upper torso and the head at ‘time zero’ of the cardiac cycle, that is, the time of the peak of the R-wave; observe the significant potential difference across the torso and arms (red versus blue surface) and a much smaller potential difference across the head. Figures 2 and 4 (*a*, green lines) show the simulated ECG waveforms—calculated using the proposed model—between the considered electrode positions on the head surface (figure 1*c*).

### 3.1 Validation of the biophysics simulations

To validate the proposed cardiac propagation model, we first employed the model to generate cardiac cycles for some well-known electrode configurations. These were the potential differences along the three standard frontal plane limb leads: Lead I: left arm (LA)–right arm (RA), Lead II: left leg (LL)–RA and Lead III: LL–LA. The so-simulated cardiac cycles for the *limb leads* are shown in figure 2, and are in very good correspondence with the real-world measurements reported in the literature [23].

### 3.2 Correspondence between simulation and measurements

Figure 3*b* shows that, due to the compromised ECG quality for the head locations, resulting from the narrow neck in particular, only one feature of the cardiac cycle, the R-wave, is visible in the raw head-ECG (see also the simulated electric potentials in figure 3*a*). However, the recorded ECG was faithful enough so that averaging of multiple cardiac cycles revealed full features of the cardiac cycle, as shown in figure 4*b* for the average cardiac cycle between two head positions.

In the second step, our biophysics simulation results for the *head leads* were compared to the real-world measurements conducted on Subject 1 (the same person for whom the simulation was performed) after processing the recordings according to algorithm 1. Here, the distinctiveness of a cardiac feature depends on two factors: (i) the maximum available potential difference on the head surface, estimated using our proposed biophysics propagation model (figure 3*a*) and (ii) the quality of the actual recorded signal (effects of electrode impedance, noise, artefacts) as shown in figure 3*b*(ii). The former is demonstrated by the model and figure 4 compares the simulation results of the proposed model (figure 4*a*) to the estimated cardiac cycles from measured data on the head (figure 4*b*). Observe that the *neck*, *jaw-centre* and *jaw-joint* channels exhibited the most pronounced cardiac features and most accurately resembled the reference ECG from the arms, while the *cheek* and *forehead* channels confirmed the expected low signal amplitudes, indicated in the simulation.

The importance of the propagation model based on the rigorous and comprehensive realistic model in figure 1 becomes more apparent when considering the, at first glance, somewhat counterintuitive results for some recording set-ups. For example, the recorded R-wave for the *forehead* channel is more pronounced when using the identified R-waves from its own (low-SNR) channel (blue line) rather than the timings from the (high-SNR) reference Lead I channel (grey line). However, our simulation also predicted a lower-amplitude R-wave (green line in figure 4*a*), in accordance with the cardiac cycle obtained using the timings from the reference ECG (grey line in figure 4*b*), thus fully supporting the empirical evidence. When the R-waves are identified in a high-noise head-ECG channel, noise peaks with a high amplitude can be incorrectly labelled as R-waves. If this happens multiple times, averaging these false high-amplitude R-waves results in an alleged R-wave in the cardiac cycle which is more pronounced than in the cardiac cycle obtained using the R-wave timings from the reference. However, as the incorrectly identified R-waves are not followed by T-waves, the T-waves across all cardiac cycles are not aligned and are therefore likely to cancel out through averaging. This is reflected in the absence of the T-wave in the right panel of figure 4*b* in the cardiac cycle obtained using the R-wave timings from the *forehead* channel (blue line). To take this effect into account when quantitatively comparing the quality of head-ECG channels, the relative amplitudes between the peaks in the cardiac cycles in head-ECG and Lead I are also considered.

Overall, compared to the standard *limb leads*, the matching between the *head leads* and Lead I was the highest. This is the case for both the simulated and measured data and was expected, because the *head leads* are approximately in parallel to Lead I and form a similar projection plane for the heart vector (figure 2*a*). Therefore, in the remainder of this study, the quality of the head-ECG will be analysed with respect to Lead I (between the arms).

### 3.3 Assessment of the performance of head-ECG

Now that we have demonstrated the theoretical and experimental feasibility of recording cardiac features from the head, a comprehensive analysis of the recorded ECG traces is next performed over five more subjects (recordings from all six subjects were previously used in [4] for a different type of analysis). For rigour, four different performance metrics were used:

(i) root-mean-square error (RMSE) between the timings of the P-, Q-, R-, S- and T-waves in the reference channel (Lead I) and a given head channel;
(ii) the Pearson correlation coefficient between the cardiac cycles obtained using the *head leads* and Lead I;
(iii) root mean square (RMS) of the ratios between the amplitude of the waves in the cardiac cycle and the amplitude of the R-wave in the same recording and channel;
(iv) RMSE between the mean cardiac cycle of a given channel and all individual cycles recorded in that same channel (based on the timing of the R-wave in the reference ECG), where the RMS was normalized by dividing by the standard deviation of the mean cardiac cycle.

The results of the four metrics are summarized in table 2 and show the means across the six subjects. Table 2 further quantitatively reinforces the findings obtained by both the simulation model and the real-world measurements shown in figure 4. The electrode locations *neck*, *jaw-centre* and *jaw-joint* consistently exhibit the most faithful cardiac features for all four applied metrics.

**Table 2.** Comparison of the cardiac cycles of the head-ECG and the reference Lead I ECG from the arms via: (i) time difference between the cardiac features, (ii) correlation of the cardiac cycles, (iii) ratio between the amplitude of the waves and the amplitude of the R-wave in a given cardiac cycle, and (iv) normalized variance. ‘ref’ denotes results for cardiac cycles for which the R-wave timings were obtained from the reference ECG (Lead I, on the arms) and ‘sig’ those for which the R-waves were obtained from the individual head channels themselves. The values represent the means across all six subjects, the first row displays the ideal values (the reference ECG compared to itself) and the highlighted row denotes the best overall performance.

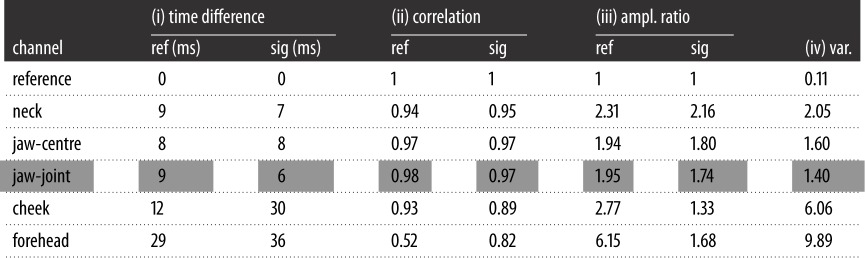

### 3.4 Head-ECG waveforms for different people

**Figure 5.**
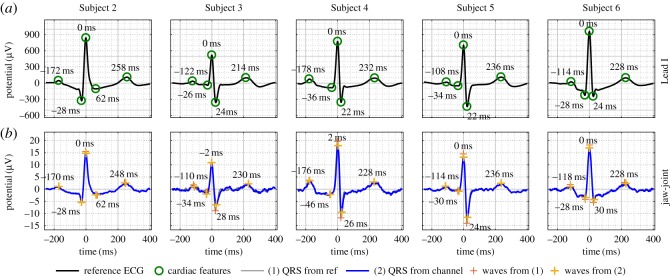
Head-ECG including the timings of the P-, Q-, R-, S- and T-waves with respect to the R-wave, as recorded from the *jaw-joints* position (*b*) benchmarked against reference Lead I arm-ECG (*a*); the amplitude in the reference ECG (from the arms) was approximately 50 times larger than the amplitude in the head channels.

While the cardiac cycles of healthy people exhibit certain common features, e.g. the P-, Q-, R-, S- and T-waves, the shapes of these characteristic waves differ between people. To establish the feasibility of recording a head-ECG cardiac cycle, the form of which is similar to the standard Lead I on the arms, we conducted experiments over multiple subjects with diverse cardiac cycles. For clarity, figure 5 shows the cardiac cycles for Subjects 2–6 for only the *jaw-joint* position, the best performing electrode position according to the metrics in table 2. The cardiac cycles obtained from the remaining recording positions are displayed in figure 8. Observe that the pattern of the QRS-complexes varies between subjects and that the head-ECG accurately resembles various patterns in the corresponding ECG recorded using Lead I.

We highlight that the timings of R-waves in the figures were not always exactly at 0 ms. This is due to different filter settings for determining the positions of the R-waves and for obtaining the full cardiac cycle (see algorithm 1). Furthermore, a low-pass filter applied to the recorded signal to remove high-frequency noise also attenuates the high-frequency component of the QRS-complex and therefore reduces the amplitude of the sharp QRS-complexes.

In head-ECG channels where the average cardiac cycles correspond to the simulated cycle when using the R-wave timings from the same channel in the averaging step, both the QRS-complexes must have been identified correctly and all the information about the different patterns in the cardiac cycle must also have been present in the given head-ECG channel under consideration. For head-ECG channels where the average cardiac cycle corresponds to the expected cardiac cycle only when R-wave timings were obtained from the reference ECG, the information about the cardiac cycle was still present in the head-ECG channel, but the noise level was too high to identify QRS-complexes correctly when using only a given individual channel.

For the two best quality head-ECG channels, the quantitative analyses indicate a slightly better performance when the timings of R-waves were taken from the reference ECG. In other words, the quality of the cardiac cycles obtained from head-ECG can be further improved through an increased accuracy of the R-wave detection, e.g. by enhancing the skin–electrode impedances or by advanced noise-reducing solutions, for example through our recently introduced co-located multimodal sensors [11,26,27]. To enhance the overall signal quality while avoiding the need for a reference ECG, an alternative way to detect QRS-complexes would be to consider all head channels simultaneously, as outlined in our recent study [2]. In this way, the accuracy in the multichannel detection of QRS-complexes from only head-ECG becomes on par with using the reference ECG.

In summary, figure 5 and table 2 show that cardiac cycles of subjects with different waveform patterns in the standard Lead I can be equally accurately obtained using head-ECG.

### 3.5 Ear-ECG waveforms

**Figure 6.**
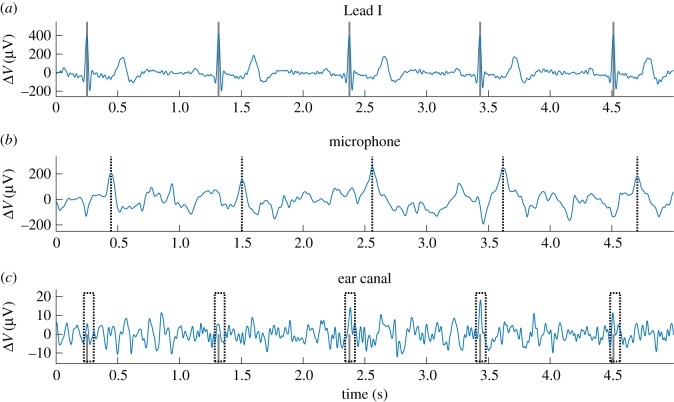
Illustration of enhanced R-waves detection in ear-ECG using a MEMS microphone co-located with the electrode [26,28]. (*a*) The standard Lead I ECG from the arms, recorded simultaneously with (*b*) the mechanical plethysmogram from an in-ear co-located MEMS microphone and (*c*) ear-ECG. The easily detectable maxima in the microphone signal (*b*) correspond to the arrival times of the pulse in the blood vessels of the ear canal and define the analysis time window for the ear-ECG signal (*c*) in which the preceding R-wave must have occurred. The timings of R-waves in Lead I are marked in the two ECG channels (*a*,*c*).

**Figure 7.**
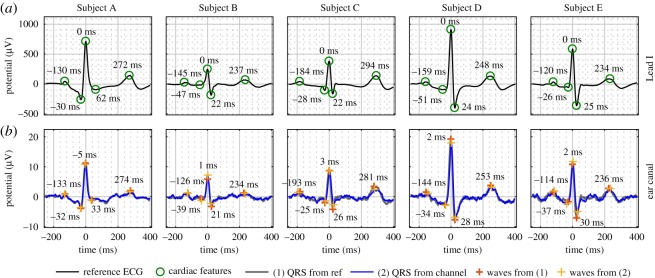
Ear-ECG including the timings of the P-, Q-, R-, S- and T-waves with respect to the R-wave, as recorded from the ear canals (*b*) and benchmarked against the reference ECG, Lead I form the arms (*a*); the amplitude in the reference (from the arms) was approximately 50 times larger than the amplitude in the head channels.

**Figure 8.**
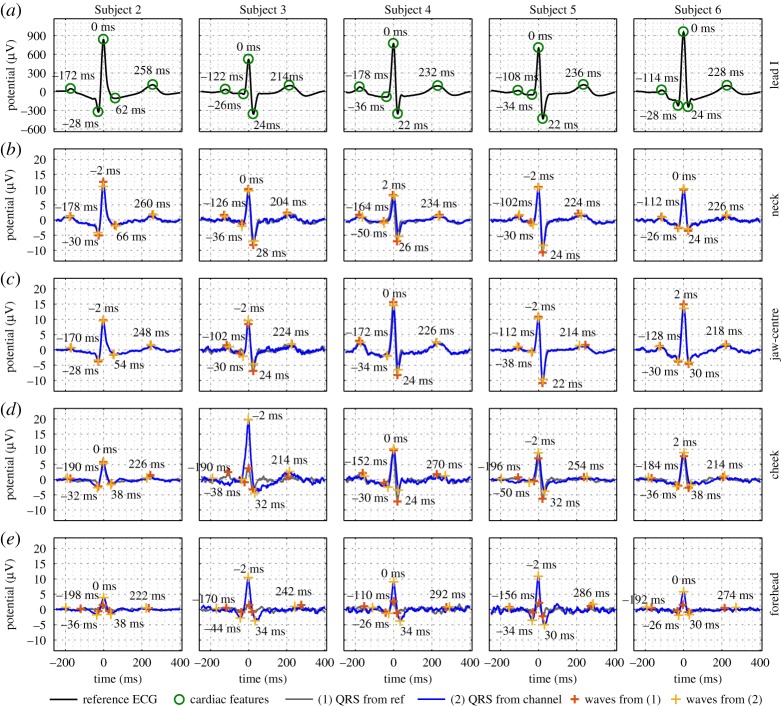
Head-ECG, including the timings of the P-, Q-, R-, S- and T-waves with respect to the R-wave, as recorded from the head positions with bipolar electrode arrangements ((*b*)–(*e*), excluding the *jaw-joints* position which is displayed in figure 5) and benchmarked against the reference ECG (*a*); the amplitude in the reference ECG from the arms was approximately 50 times larger than the amplitude in the head channels.

**Figure 9.**
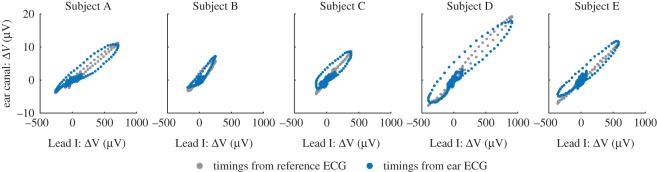
The agreement between the cardiac cycles obtained from the ear canal (ear-ECG) and Lead I (standard ECG). All points on a straight line would indicate a perfect match. The grey points represent the results when using the reference Lead I ECG to identify the timings of R-waves, and the blue points the results when using the ear-ECG channels only; the amplitude in the reference (Lead I from the arms) was approximately 50 times larger than the amplitude in the ear channels. Observe an excellent agreement between ear-ECG and Lead I ECG, as indicated by a small deviation of the points from a straight line.

The inner ear location is more convenient for health monitoring than helmet-worn sensors, as it enables unobtrusive recording of vital signs and EEG traces in a wider range of scenarios. To establish the feasibility of ECG recordings from within the ear canal (ear-ECG), we used our ‘hearables’ earpiece with embedded sensors, with electrodes made from conductive fabric and microphones [11,26,28], and recorded 4-minute segments of cardiac signals from five subjects. The data were processed according to algorithm 1 while the measurements from the microphones embedded in the earpieces were used to support the identification of the timings of QRS-complexes, as they are capable of detecting the tiny pulsations of blood vessels in the ear canal. This approach is based on the delay between the peak of the R-wave and the maximum in the mechanical measurement from the microphone [29]. Therefore, the R-wave detection can be limited to the interval between 220 ms and 140 ms before the occurrence of a peak in the microphone signal, as shown in figure 6. In the simultaneous microphone and ear-ECG recordings it is possible to calculate the pulse wave velocity and the pulse waveform. Both are indicators for arterial stiffness, which itself is an indicator for cardiovascular diseases. Figure 7 shows the high correspondence between the cardiac cycles recorded from the ear canals and the reference (Lead I from the arms). Observe that the differences in the characteristic waveforms in the cardiac cycle in several recordings were correctly determined using ear-ECG. Additionally, the ratio between the amplitude of the R-wave peak in Lead I and the R-wave peak in ear-ECG was approximately 50 across all subjects. In figure 9, the agreement between the cardiac cycles as recorded from Lead I and the ear canal is analysed visually and in table 3 quantitatively.

**Table 3.** Comparison of the cardiac cycles of the ear-ECG and the reference Lead I ECG from the arms via: (i) time difference between the cardiac features, (ii) correlation of the cardiac cycles, (iii) ratio between the amplitude of the waves and the amplitude of the R-wave in a given cycle, and (iv) normalized variance. ‘ref’ denotes results for cardiac cycles for which the R-wave timings were obtained from the reference ECG (Lead I, on the arms), and ‘sig’ those for which the R-waves were obtained from the ear channel itself. The values represent the means across all five subjects and the first row displays the ideal values (the reference ECG compared to itself).

	(i) time difference		(ii) correlation		(iii) ampl. ratio		
channel	ref (ms)	sig (ms)	ref	sig	ref	sig	(iv) var.
reference	0	0	1	1	1	1	0.22
ear canal	8	9	0.96	0.90	1.57	1.43	2.31

## 4. Conclusion

We have introduced a rigorous biophysics model of the propagation of electric potentials from the heart to the head, and have simulated the shape and timing of cardiac cycles at a number of locations on the head surface and the ear canal. This has been achieved through creating realistically shaped body geometries and by accounting for dielectric properties of body tissues along the propagation path from the heart to the head. In this way, we have identified the electrode locations on the head for which the shape and timing of ECG features are minimally distorted, a finding which has been supported by real-world head-surface ECG recordings. In addition, the head- and ear-ECG have been comprehensively validated against standard Lead I ECG (reference ECG from the arms), through the respective relative timing of the characteristic waves in ECG (relative to the R-wave), their correlation and amplitude ratios between the characteristic waves. Despite the lower SNR of head-surface and in-ear measurements, we have demonstrated the feasibility of detecting and measuring all components of the cardiac cycle from several head and in-ear positions. In this way, we have established a theoretical and experimental foundation for ECG recordings from head-worn health-monitoring devices, such as the smart helmet and wearable in-ear devices, the so-called ‘hearables’. Future work will consider practical aspects in long-term wearable scenarios, such as different noise sources and artefacts, and various cardiac conditions.

## Data accessibility

The data and scripts have been uploaded to Dryad. They can be accessed here: https://doi.org/10.5061/dryad.3n08c [30].

## Appendix A

For completeness, figure 8 shows the cardiac cycles for Subjects 2–6 from the remaining four head channels (those not considered in figure 5), compared to the reference channel Lead I from the arms. The visual inspection of the matching between the head ECG and the reference ECG corresponds to the values in table 2; the channels *neck* and *jaw-centre* exhibit the best results of the remaining four channels.
